# Dynamic 3D Measurement without Motion Artifacts Based on Feature Compensation

**DOI:** 10.3390/s23167147

**Published:** 2023-08-13

**Authors:** Guoce Hu, Jun Wang, Huaxia Deng, Mengchao Ma, Xiang Zhong

**Affiliations:** 1 School of Instrument Science and Opto-Electronics Engineering, Hefei University of Technology, Hefei 230009, China; huguoce@126.com (G.H.); mmchao@hfut.edu.cn (M.M.); zhx0325@hfut.edu.cn (X.Z.); 2 School of Mechanical Engineering, Anhui University of Science and Technology, Huainan 232001, China; jjwang@aust.edu.cn; 3 CAS Key Laboratory of Mechanical Behavior and Design of Materials, Department of Modern Mechanics, University of Science and Technology of China, Hefei 230027, China

**Keywords:** phase-shift profilometry, error compensation, motion artifacts, three-dimensional reconstruction

## Abstract

Phase-shift profilometry (PSP) holds great promise for high-precision 3D shape measurements. However, in the case of measuring moving objects, as PSP requires multiple images to calculate the phase, the movement of the object causes artifacts in the measurement, which in turn has a significant impact on the accuracy of the 3D surface measurement. Therefore, we propose a method to reduce motion artifacts using feature information in the image and simulate it using the six-step term shift method as a case study. The simulation results show that the phase of the object is greatly affected when the object is in motion and that the phase shift due to motion can be effectively reduced using this method. Finally, artifact optimization was carried out by way of specific copper tube vibration experiments at a measurement frequency of 320 Hz. The experimental results prove that the method is well implemented.

## 1. Introduction

Due to various benefits like excellent precision, fast measurement in multiple dimensions, and the ability to automate the process, non-contact optical 3D shape measurement technology has gained popularity [1,2,3,4]. This technology has been extensively researched and applied in various fields under computer control constraints [5,6,7]. One of the most widely used methods for measuring optical 3D structures is fringe projection profilometry (FPP), which relies on phase calculation. FPP is well-known for its accurate measurements and high spatial resolution [8,9]. In FPP technology, Fourier transform profilometry (FTP) and phase-shifting profilometry (PSP) have gained significant research value in recent years [10,11]. FTP is a single-frame raster projection method based on spatial filtering, which requires only one image to reconstruct the target information [12]. Spectrum aliasing can affect the accuracy of 3D reconstruction [13,14]. When studying object motion, FTP may not meet the requirements for object reconstruction accuracy [15,16]. On the other hand, PSP is the most widely studied method and can obtain high robustness and high-precision pixel-wise phase unwrapping [17,18]. However, PSP may encounter difficulties when dealing with scenes that have dynamic motion, as the movement of objects can cause distortion in the phase, leading to errors. This issue is particularly prominent when the motion of the object between inter-frame times is significant [19,20]. To address these issues, research on dynamic 3D shape measurement using PSP has focused on reducing the number of patterns required for each 3D reconstruction and improving the quality of the measurement to reduce motion artifacts.

The enhancement of measurement efficiency in Fourier transform profilometry (FPP) can be achieved by optimizing the technique of reducing the number of projections, as proposed by various researchers [21,22,23]. Nonetheless, this approach may result in phase ambiguity due to the periodicity of the sinusoidal signal [24]. One possible solution is to utilize the time phase unwrapping (TPU) algorithm with auxiliary patterns such as Gray codes or multi-wavelength fringe [25,26]. Another approach is the use of composite phase-shifting schemes like dual-frequency PSP, which can resolve phase ambiguity without significantly increasing the number of patterns. However, PSP requires at least three fringe patterns to achieve high-precision pixel-wise phase measurement [27,28]. These methods often compromise measurement accuracy by relying on low-frequency fringes for reliable phase unwrapping. Therefore, improving measurement accuracy is still a major challenge in dynamic 3D shape measurement using FPP.

Numerous studies have been conducted with the aim of improving the precision of measurement and reducing motion artifacts in FPP. Weise et al. (2007) introduced a method based on least squares fitting to estimate the phase shifts caused by movement [29]. Pistellato (2019) introduced a probabilistic framework aimed at mitigating the influence of errors. Yet, this framework does not address errors caused by motion artifacts [30,31]. Lu (2016) developed an iterative algorithm utilizing least squares to correct the unknown phase offset induced by the 3D rigid motion of the object [32]. Zuo (2018) summarized the methods affecting the development of projection measurements and also proposed a strategy to reduce motion errors in three-step projection measurements [33]. These techniques assume that each pixel undergoes uniform motion, which may not be valid for objects exhibiting different motion patterns. Wang et al. (2021) proposed a four-step phase-shifting contour method that compensates for error by utilizing the intermediate phase of two results [34]. This method is effective in the presence of uniform motion but fails to handle non-uniform and non-rigid objects. Wang (2018) utilized the Hilbert transform to alleviate motion error, which is effective for periodic motion [35]. Nevertheless, current methods still face challenges in dealing with motion artifacts, particularly in scenarios where the target object exhibits high moving frequency or large moving amplitude.

In this paper, a new technique is introduced to suppress motion-induced artifacts in the reconstruction of 3D point clouds of free-moving objects, thereby improving the accuracy of 3D measurements. The proposed algorithm is a new feature phase optimization algorithm that reduces motion errors and optimizes the artifacts of the non-periodic motion of flexible copper tubes, and the feasibility and effectiveness of the method is verified by simulation and experiment. In the simulation, this study uses a hemispherical object with a size of 512 × 512 pixels to test our artifact suppression algorithm. In experiments, this study uses structured light projection to measure the free motion of a copper tube under the impact of a small hammer. In a supplementary experiment, we measure the 3D point cloud data of a freely falling ping-pong ball bouncing back after hitting the ground, and optimize the results. Finally, our method is compared with other optimization algorithms for periodic errors and its superior performance in reducing reconstruction artifacts is demonstrated.

The rest of the paper is organized as follows: Section 2 illustrates the principles of the 3D measurement method for reducing motion-induced errors and simulates the associated experimental effects. Section 3 presents some experimental results to validate the proposed method. Section 4 summarizes and discusses the feature of the proposed method.

## 2. Principle

### 2.1. Motion-Induced Error for Six-Step Phase-Shifting Method

For a generic N-step phase-shifting algorithm, the intensity distribution of the Nth stripe can be described as:

(1) $$I_{n}\left(x,y\right)=A\left(x,y\right)+B\left(x,y\right)cos\left[\Phi \left(x,y\right)+2\pi \left(n-1\right)/N\right],$$

where A(x,y), B(x,y), and Φ(x,y) denote the average intensity, intensity modulation, and phase map, respectively. $I_{n}\left(x,y\right)$, *n* = 1, 2, *…*, *N*, *n* is the intensity recorded by the camera.

In dynamic measurements, according to the sampling theorem, it is hoped that the fewer projection patterns the better, but the number of projection patterns is equally related to the quality of the reconstructed phase. Therefore, in order to balance the effect of dynamic measurements and the quality of the reconstructed phase, this paper takes the six-step projection measurement algorithm as an example for error calculation and compensation.

For a standard six-step phase-shifting method with π/2 phase shift, the wrapped phase can be computed using the following equation:

(2) $$\Phi \left(x,y\right)=\tan^{-1}\left[\frac{\sqrt{3}\left(I_{2}+I_{3}-I_{5}-I_{6}\right)}{2I_{1}+I_{2}-I_{3}-2I_{4}-I_{5}+I_{6}}\right].$$

The N-step phase-shifting algorithm can obtain an accurate phase map Φ(x,y) if the phase shift 2π(n−1)/N is precise. If the measured object is moving, the phase shift at each pixel in the captured images will have an additional unknown phase-shift error $\varepsilon_{n}\left(x,y\right)$ [*n* = 1, 2, 3, *…*, *N*−1] due to the object’s motion. The error $\varepsilon_{n}\left(x,y\right)$ will result in pattern distortion and phase calculation as follows:

(3) $$\left\{\begin{array}{c} I{}_{1}^{\prime }\left(x,y\right)=A\left(x,y\right)+B\left(x,y\right)cos\left[\Phi \left(x,y\right)\right]\\ I{}_{2}^{\prime }\left(x,y\right)=A\left(x,y\right)+B\left(x,y\right)cos\left[\Phi \left(x,y\right)+1/3\pi +\varepsilon_{2}\left(x,y\right)\right]\\ I{}_{3}^{\prime }\left(x,y\right)=A\left(x,y\right)+B\left(x,y\right)cos\left[\Phi \left(x,y\right)+2/3\pi +\varepsilon_{3}\left(x,y\right)\right]\\ I{}_{4}^{\prime }\left(x,y\right)=A\left(x,y\right)+B\left(x,y\right)cos\left[\Phi \left(x,y\right)+\pi +\varepsilon_{4}\left(x,y\right)\right]\\ I{}_{5}^{\prime }\left(x,y\right)=A\left(x,y\right)+B\left(x,y\right)cos\left[\Phi \left(x,y\right)+4/3\pi +\varepsilon_{5}\left(x,y\right)\right]\\ I{}_{6}^{\prime }\left(x,y\right)=A\left(x,y\right)+B\left(x,y\right)cos\left[\Phi \left(x,y\right)+5/3\pi +\varepsilon_{6}\left(x,y\right)\right]. \end{array}\right.$$

For a small phase-shift error ε, sin(ε) ≈ε and cos(ε) ≈ 1. According to the phase affected by the phase shift error, $\varepsilon_{n}$ can be expressed as:

(4) $$\left\{\begin{array}{c} \phi {}^{\prime }\left(x,y\right)\approx \tan^{-1}\left[\frac{\sqrt{3}\left(A\cos\phi +B\sin\phi \right)}{C\cos\phi +D\sin\phi }\right]\\ A=\frac{1}{2}-\sqrt{3}-\frac{1}{2}\varepsilon_{3}-\frac{1}{2}\varepsilon_{6}\\ B=-1-\frac{\sqrt{3}}{2}\varepsilon_{2}-\frac{\sqrt{3}}{2}\varepsilon_{3}-\frac{\sqrt{3}}{2}\varepsilon_{5}+\frac{1}{2}\varepsilon 5-\frac{\sqrt{3}}{2}\varepsilon_{6}\\ C=4+\frac{1}{2}+\sqrt{3}+\frac{\sqrt{3}}{2}\varepsilon_{3}-\frac{\sqrt{3}}{2}\varepsilon_{5}+\frac{1}{2}\varepsilon_{6}\\ D=\frac{1}{2}-\frac{1}{2}\varepsilon_{2}-\frac{\sqrt{3}}{2}\varepsilon_{2}-\frac{1}{2}\varepsilon_{3}-2\varepsilon_{4}-\frac{1}{2}\varepsilon_{5}-\frac{\sqrt{3}}{2}\varepsilon_{6}. \end{array}\right.$$

Here, the relationship between each error and the actual phase is calculated. It can be seen from the formula that, if the error is different every time, the actual phase is related to the error generated by each projection. In order to reduce the motion error, local feature matching is used to calculate the displacement changes in the six-step item movement, and then the motion is compensated to reduce the generation of artifacts.

### 2.2. Local Feature Matching

**Figure 1:**
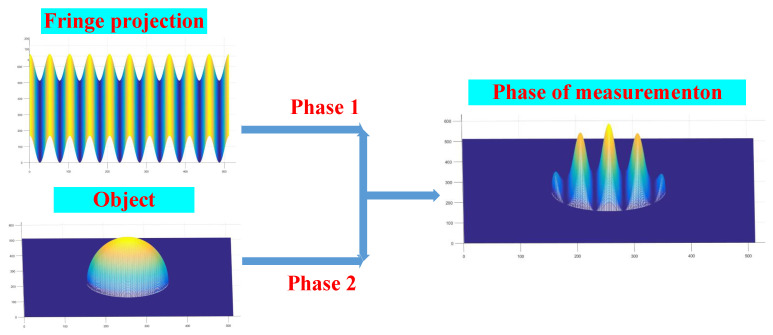
The influence of phase.

The measured phase consists of the phase 2 of the object itself and the projected phase 1, and its gray value is also related to them. When motion occurs, it affects the change in grey value corresponding to the projection phase. Such as in Figure 1.

**Figure 2:**
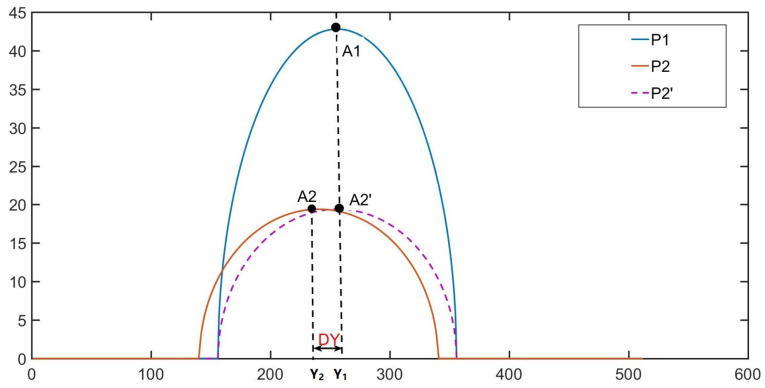
Phase error optimization.

The projections at different moments are affected by the projection fringes when the effect of the motion error of the object is taken into account, as shown in Figure 2.

In vertical projection measurement, the phase obtained in the first projection is $P_{1}$, and the phase obtained in the second projection is $P_{2}$. Since $P_{2}$ is generated by the movement of the object, if the object is not moving in the second projection, the phase should be $P_{2}^{\prime }$. The difference between the two phases is actually a DY shift in the Y-direction, which corresponds to the maximum value (or minimum value) of the object’s features: A1 should be the maximum value of phase $P_{1}$, and its corresponding coordinate is $Y_{1}$; A2 is the maximum value of phase $P_{2}$, and its corresponding coordinate is $Y_{2}$; the optimized phase $P_{2}^{\prime }$ can be obtained by the following equation:

(5) $$P_{2}^{\prime }=P_{2}+\left(Y_{1}-Y_{2}\right).$$

The above is the phase optimization of a single column, while the phase optimization of a graph can be carried out using the following equation:

(6) $$\sum_{x}^{n}P_{2}^{\prime }=\sum_{x}^{n}\left(P_{2}+Y_{1}^{n}-Y_{2}^{n}\right).$$

$Y_{2}^{n}$ in the formula represents the feature phase position of the second image in the *n* row, and $Y_{1}^{n}$ represents the feature phase position of the first image in the *n* row as the main feature. The optimized phase $P_{2}^{\prime }$ can be found by the above equation, and the same equation can be extended to the third to the sixth amplitude as follows:

(7) $$P_{n}^{\prime }\left(x,y\right)=\sum_{x}^{n}P_{i}^{\prime }=\sum_{x}^{n}\left(P_{i}+Y_{1}^{n}-Y_{i}^{n}\right).$$

$P_{n}^{\prime }\left(x,y\right)$ represents the optimized phase, where *n* represents the first image. $Y_{i}^{n}$ represents the Y coordinate of its corresponding feature location and this parameter is used to calculate the compensated pixel value of the image. The main features chosen in the above equation are obtained from the phase of the 1st image, so all the subsequent 2–6 images are optimized for the phase values by the features of the 1st image, which removes the phase effect of the motion on the projection measurements, and ultimately optimizes the point cloud data for better results.

### 2.3. Simulation

**Figure 3:**
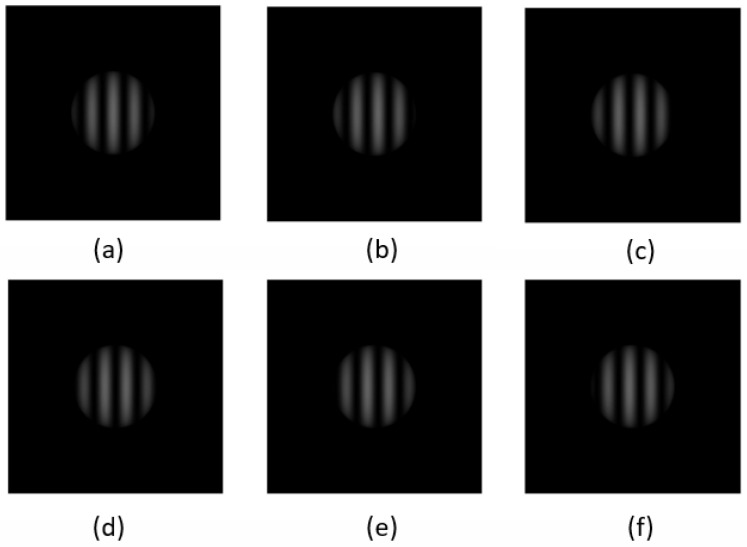
Simulation diagram of six-step phase. (**a**–**f**) are the phases of the projection grating compounded to the object at intervals of 1/6 π respectively.

In order to implement the present method, the validation is continued here using simulation. Firstly, a picture with 512 × 512 pixels and a grating width of 51 pixels is designed in the simulation experiment, and a hemispherical shape with a pixel size of 100 is designed in the center of the picture for simulation. The period of the grating is the pixel size of one side divided by 10, which satisfies the design in our actual measurement train of thought. The experimental diagram after simulation is shown in Figure 3.

The first set of experiments was performed in order to verify the artifacts produced by uniform motion and their elimination. On this basis, the position of the object is moved. The specific moving value is that each picture moves in the X direction of 5.5 sub-pixels, and six pictures are regenerated and phase decoded.

**Figure 4:**
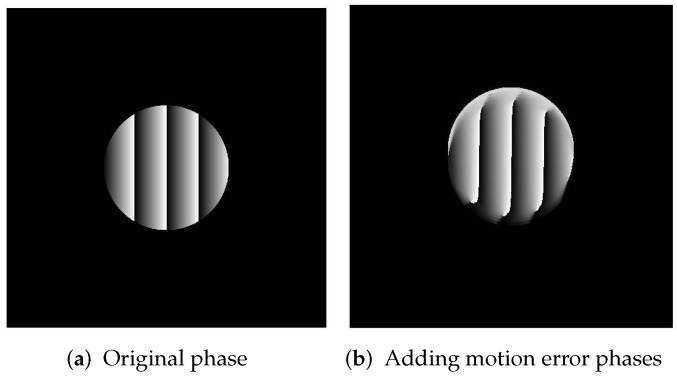
Comparative simulation of actual phase and error phase.

As shown in Figure 4, the left side is the phase information obtained when the object is not moving, and the right side is the phase information with artifacts obtained by the object under the influence of motion error.

**Figure 5:**
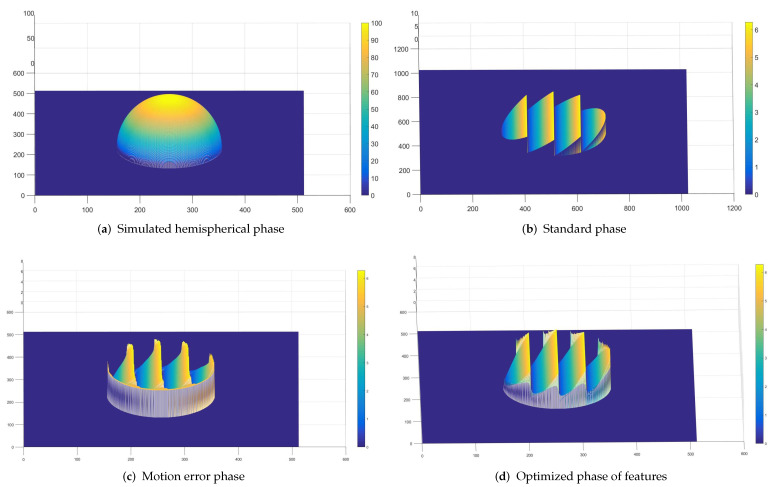
Standard phase and comparison phase before and after optimization.

As shown in Figure 5a, the phase of the 100 × 100 pixel hemisphere of the simulation is used. The phase of the projection is obtained without considering errors, as shown in Figure 5b. The added error is an offset of 5.5 pixels in each image starting from the second image, and the result is shown in Figure 5c. Figure 5d shows the result after optimization using the method proposed in this paper. From the figure, it can be seen that, before optimization, the phase of the object produces displacement artifacts in the Y direction. This phase result has a significant impact on the reconstruction of the target. The optimized phase is closer to the true phase. To this end, we extracted the phase value of the target object at 250 pixels and compared it with the actual value. The results among the three are as follows.

**Figure 6:**
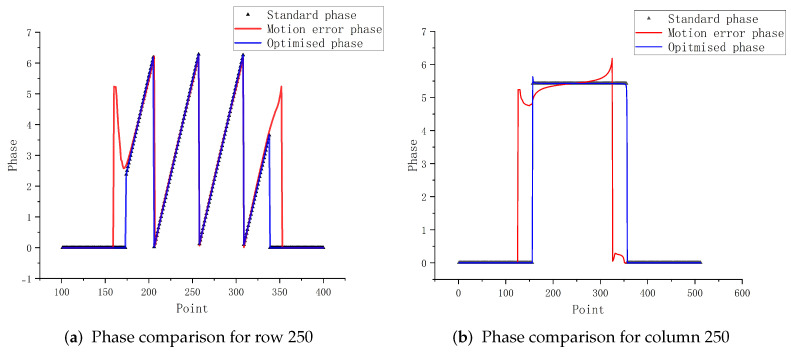
Contrasting phases of uniform motion.

As can be seen from Figure 6, at an x-coordinate of 250 pixels, the phase values of the object show a periodic variation, in line with the 2π law of motion; while the motion errors produce phase values with a large discrepancy, the optimized data are obviously better than the data before optimization. The standard picture presents a phase change from 150 to 350, and other parts remain stable. The optimized phase also conforms to this feature, but there are some errors at the change, which should be caused by the fact that the optimized parameters cannot achieve sub-pixel level matching (in the simulation experiments to verify the irregular effects of motion, the motion parameters of all six images are different, where there is also the motion of 0.5 pixels of object motion).

The second set of experiments had exactly the same parameters as the first set of experiments, with the difference that the position of the moving pixels was not uniform, moving (7.8, 10.2, −2.5, −6, 4.3) pixels from frame 2 to frame 6, respectively.

**Figure 7:**
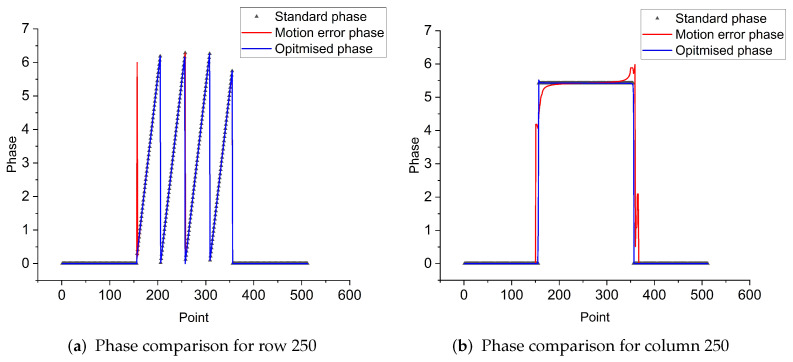
Contrasting phases of nonuniform motion.

From the above figure, it can be seen that the effect on the phase per column is greater than the effect on the phase of rows in non-uniform motion. The comparison of Figure 6 and Figure 7 shows that the error of its column phase will be more obvious in non-uniform motion. The optimization method in this paper has good results for both uniform and non-uniform motion objects.

### 2.4. Decoding and Reconstruction

**Figure 8:**
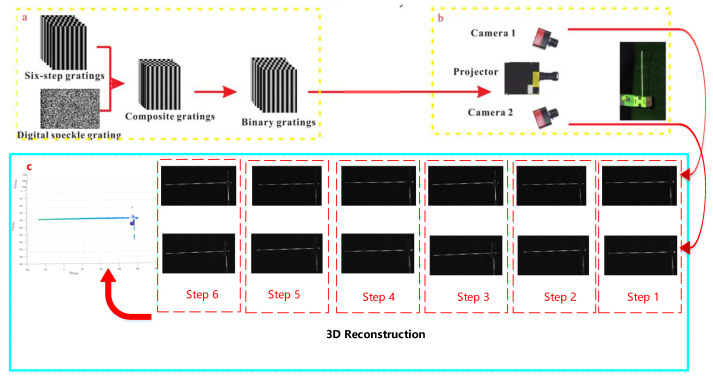
Schematic diagram of experiment.(**a**) indicates that the composite grating consists of a six-step projection grating and a scattering grating, and (**b**) is the projection of the composite grating in (**a**) onto the surface of the object and the acquisition of the image to (**c**). (**c**) represents the 3D point cloud image of the copper tube obtained by decoding the six images.

The details of the experiment are shown in Figure 8. The first step is to project a combination of stripes and scatter onto the object, then generate six term-shift images from the measured object, and then compensate for the phase of the motion by means of feature optimization. After the wrapped phase is obtained, the speckle image is embedded into the fringe pattern to solve the phase blur problem. Combining the six-step phase-shift grating and the binary digital speckle image into a composite grating, and directly adding the gray value of the speckle image to the average gray value of the phase-shift grating:

(8) $$H_{n}\left(x,y\right)=I_{n}\left(x,y\right)+S\left(x,y\right),$$

where *n* is the number of fringe patterns from 1 to 6, $I_{n}\left(x,y\right)$ represents the six-step phase-shift fringe pattern, and S(x,y) is the digital speckle image. Embedding the digital speckle image into the phase-shift fringe pattern means that the phase correlation and digital correlation can be combined.

## 3. Experiments

To evaluate the effectiveness of the newly proposed method for compensating for motion-induced errors in six-step phase-shifting profilometry, we developed an experimental measurement system. This system consisted of two camera with an imaging resolution of 1280 × 1024 pixels and two 12mm imaging lens. This experiment used a digital projector (LightCrafter 4500, from Texas Instruments Incorporated, Dallas, TX, USA) with a resolution of 912 × 1140 pixels. To ensure synchronization between the camera and projector, the camera was triggered by a signal from the projector. In this experiment, the projection and capturing rates were set to 320 frames per second. For the measurements, we focused on a 10 mm copper pipe, which was vibrated by tapping on it with a hammer. This vibration simulates the free vibration of a copper tube, where one end of the tube is clamped and fixed to a vibration table.

**Figure 9:**
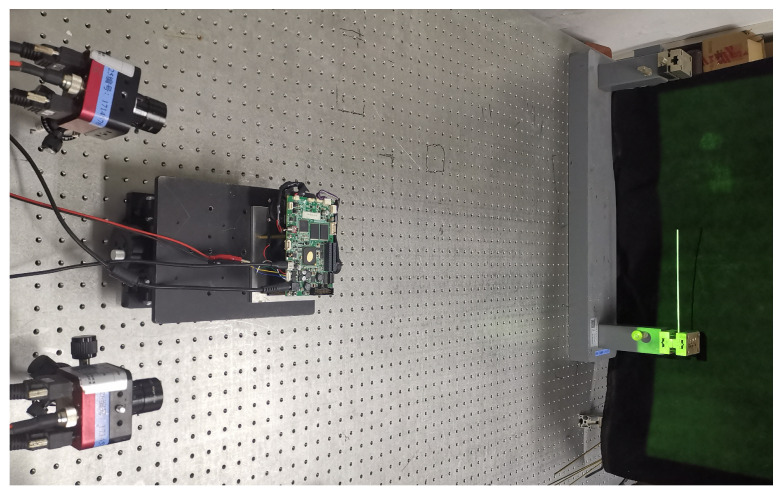
Experimental test chart.

The setup for the experiment is shown in the diagram in Figure 9. The calibration of the left and right cameras was first performed to calibrate the internal and external parameters of both cameras. Then the composite stripes were projected onto the copper tube by means of a projector and the vibration was realized by striking the tube with a small hammer. Finally, the camera was triggered by the projector to collect the pictures of the time series of the copper tube. The experimental pictures obtained from the left and right cameras were then used to create a 3D point cloud.

In order to verify the effectiveness of this method, we selected the six-step term shift method to test when the motion amplitude changes greatly. Six consecutive images were selected from the acquired data for phase reconstruction, and the results are as follows.

**Figure 10:**
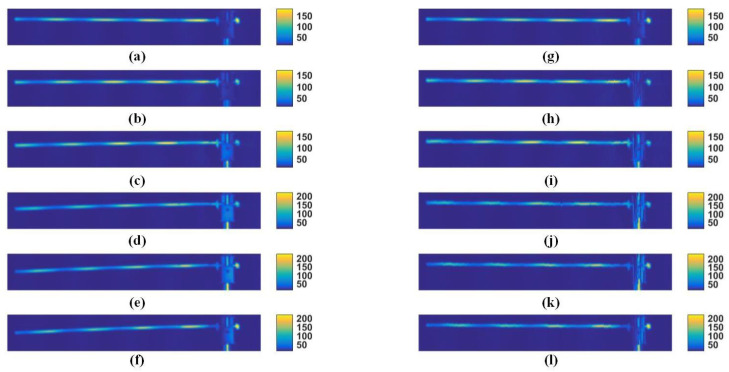
Gray scale of the image of a copper tube in a six-step projection. Where (**a**–**f**) are the images taken by the camera for the projections of steps 1–6, and (**g**–**l**) are the images of the above (**a**–**f**) optimized by the method in this paper.

Figure 10a–f show the results taken by the camera, where the color corresponds to the grayscale value of the place. Figure 10g–l correspond to the image on the left optimized by the feature algorithm of this paper, respectively. From the comparison in the figure, it can be seen that, the closer to the left side of the image, the greater its movement will be, and the corresponding optimized effect will be more obvious.

The parcel phases are obtained from these 6 images separately and the results and comparisons are as follows.

**Figure 11:**
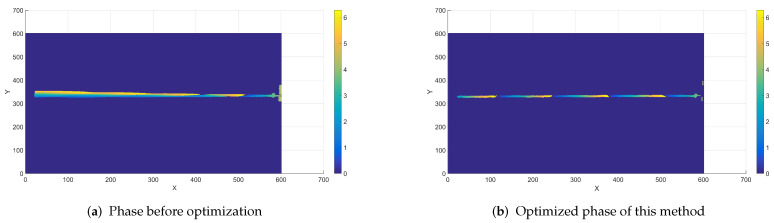
Compare measured results with optimized results.

The comparison of phase results shows that the optimized algorithm in this paper has obvious effect of eliminating the influence of motion in the phase. In order to highlight the advantages of this scheme, this paper compares and discusses the reconstruction results of other schemes. As can be seen from the Figure 11, the phase obtained by the six-step term shift method is affected by the motion in the case of motion, resulting in a term shift in the Y direction of the image, which results in great error. However, after the motion is compensated for by the method in this paper, its motion phase is compensated and, as a result, there is basically no significant artifact in the phase range. It can be seen that the method in this paper has a good effect. Select the main axis of the pipeline, extract the phase of this point, and compare it with Wang’s method [35] to obtain the following figure.

**Figure 12:**
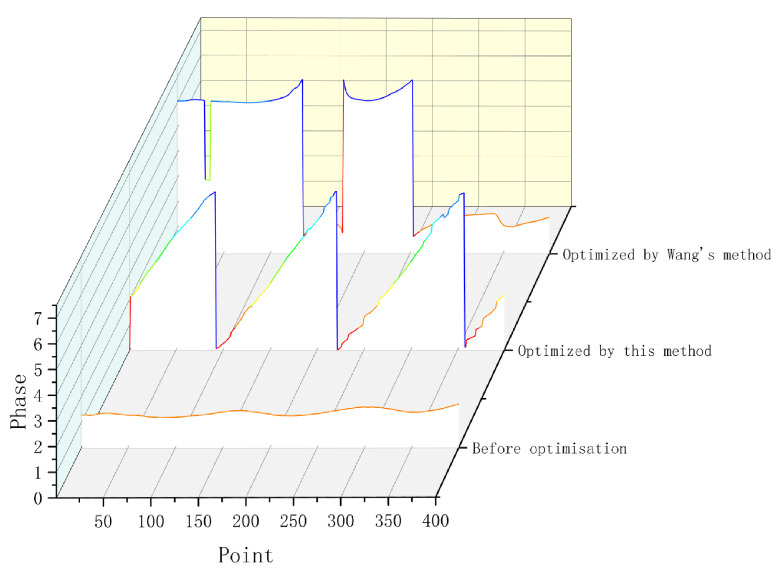
Phase result comparison.

As shown in Figure 12, the phase information in the image with pixel coordinates of 398 is selected. The Y-direction represents the phase value of the image, and its range is in [0,2π]. It can be seen from the figure that the phase cannot be reconstructed without any method, and the phase information is not obvious. After optimization using Wang’s method, some extreme changes of the phase occurred, and some wrong phase information was obtained. Only when the method in this paper is used for optimization can the phase information be clearly distinguished and the phase reconstruction be carried out. By matching the phases reconstructed by the left and right cameras, the three-dimensional point cloud images of these 6 images at a single moment are obtained.

**Figure 13:**
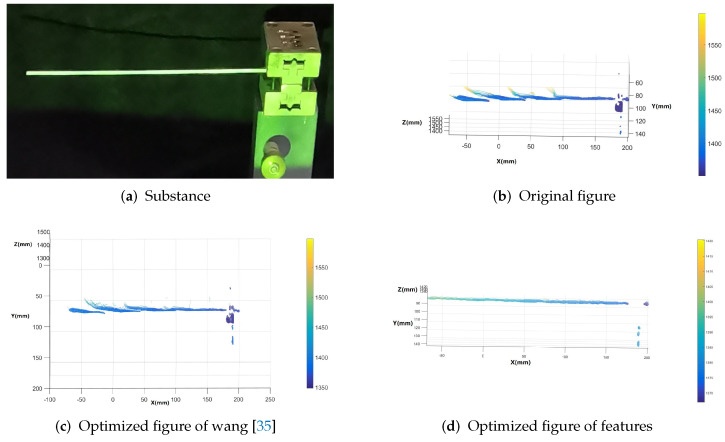
Comparison of the reconstruction results.

From the coordinates of the three reconstructed point clouds, as shown in Figure 13, it can be seen that the three-dimensional effect reconstructed from the original phase produces a large-area fracture effect, which makes a copper tube that should have been continuous produce a discontinuous point cloud image. When Wang’s method is optimized, there is also a fracture, and some point cloud coordinates are distorted. For the method in this paper, the three-dimensional point cloud reconstructed by the optimized phase can be in the same coordinate system continuously, and the result can be regarded as reliable.

### Free-Fall Experiment

**Figure 14:**
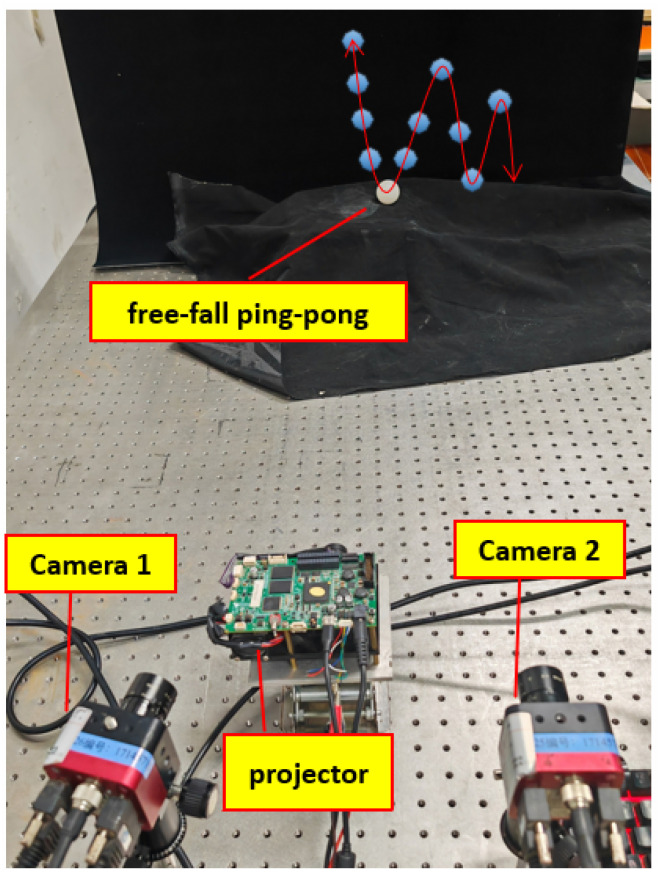
Balloon experiment.

In order to verify that this experimental method has a stable optimization effect, a free-falling ball is selected for motion artifact elimination experiments in this paper. As shown in Figure 14, the ball free-falls in the air through the ground, rebounds, and then continues to fall freely. Due to the influence of gravity, the process of the ball falling and bouncing is realized as non-uniform free motion.

Due to the fast falling process of the ball, the sampling frequency of the camera is still set to 256 Hz, so that the motion trajectory of the ball landing and bouncing can be captured as much as possible. The results of reconstructing the phase and 3D point cloud after selecting a certain 6-frame image of the ball after bouncing are as follows.

**Figure 15:**
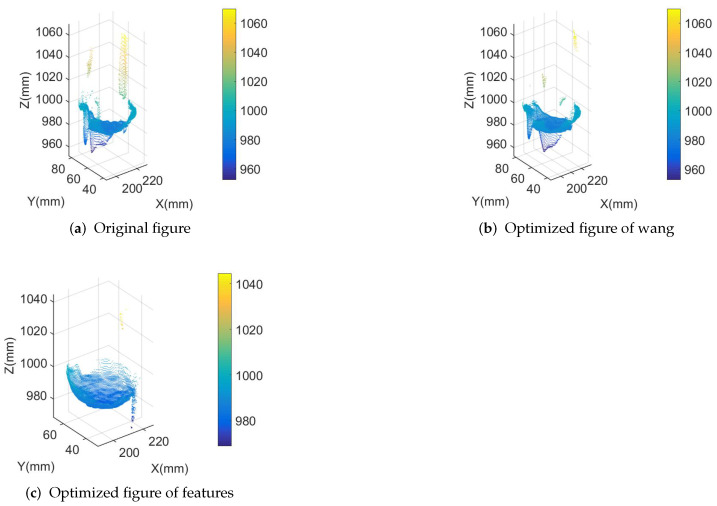
Comparison of reconstruction results for small balls.

From analyzing the coordinates of the three reconstructed point clouds depicted in Figure 15, it is evident that the 3D point cloud derived from the original phase does not exhibit a spherical shape. This distortion is a result of the ball’s movement during its ascent. A similar distortion can be observed when applying Wang’s method as shown in Figure 15b. However, the 3D point cloud generated by the method proposed in this paper clearly demonstrates a spherical surface. This serves as strong evidence that the results obtained by this method are trustworthy.

By fitting their point cloud, spherical results can be obtained as shown in Table 1.

The table compares the standard deviation and mean distance of the three sets of point clouds fitted to the blob. When the point cloud generated from the original data is used, its standard deviation reaches 6.675 mm, and when it is optimized using the features proposed in this paper, the standard deviation is reduced to 1.176 mm, which is much better than the original results. Better still, the optimized 3D point cloud in this paper fits the sphere with an average distance of 0.245 mm.

## 4. Conclusions

The PSP method is used to measure dynamic scenes where the motion of the object between frames is not negligible, in which case it will lead to phase errors and thus motion artifacts. This paper proposes a method to eliminate motion artifacts by using the information of object features, using the information of one frame of the object as a basis upon which to phase optimize the other frames.This method has the following advantages over other methods:

- The method in this paper addresses the effect of motion artifacts in the case of motion, and the method is able to optimize the measurement results for situations in which the amplitude of motion is large.
- Compared to the effects of motion errors arising from other targeted periodic moving objects, this paper targets flexible copper tubes with non-uniform velocities and the solution in this paper offers better results in terms of optimization.
- Compared to most other cases where only 60 Hz object motion is achieved, this paper achieves a reconstruction of the 3D point cloud at 320 Hz, which is more suitable in terms of frequency for the scenario of engineering applications.

Despite these advantages, the proposed method has some limitations. The first is that this paper is aimed at objects with simple textures; in the case of objects with complex textures, the feature phases do not match well and are prone to mis-matching. Secondly, the optimization method in this paper does not achieve sub-pixel level optimization, so there are still local errors in the optimized phases, as shown in Figure 13. Therefore, in the future, we will incorporate more effective methods, such as machine learning, in the hope of further improving its application in dynamic measurements.

## Figures and Tables

**Table 1:** Three-dimensional (3D) point cloud spherical fitting.

Experimental Methods	Standard Deviation (mm)	Average Distance (mm)
Primordial point cloud	6.675	1.833
Optimized of Wang’s method	5.422	1.352
Optimized of features method	1.176	0.245

## Data Availability

Not applicable.
